# Can Early Diagnosis of Varices, Regular Praziquantel, and Reduction of Hepatitis Coinfection Reduce Mortality among Patients Attended for Periportal Fibrosis in Northwestern Tanzania? A Case-Control Study

**DOI:** 10.1155/2020/5484315

**Published:** 2020-03-13

**Authors:** Daniel W. Gunda, Elizabeth F. Mtui, Semvua B. Kilonzo, Paulina M. Manyiri, David C. Majinge, Humphrey D. Mazigo

**Affiliations:** ^1^Department of Medicine, Catholic University of Health and Allied Sciences, 1464 Mwanza, Tanzania; ^2^Department of Medicine, Bugando Medical Centre, 1370 Mwanza, Tanzania; ^3^Department of Parasitology, Catholic University of Health and Allied Sciences, 1464 Mwanza, Tanzania

## Abstract

**Background:**

*Schistosoma mansoni* is highly endemic in the Lake Zone part of Tanzania and most people are chronically infected. Periportal fibrosis (PPF) is the commonest complication of chronic *S. mansoni* infection documented in up to 42% of studied participants in the community-based studies. These patients are at high risk of mortality since most of them are diagnosed late with bleeding varices. At Bugando, Schistosoma-related varices contributed to 70% of patients admitted due to vomiting blood with a two months' mortality of over 10%. Earlier studies had reported higher mortality of up to 29% among patients with PPF even with the best in-hospital care. Understanding factors that increased the risk of mortality is important clinically in devising ways that can improve the outcome of this subgroup of patients.

**Methods:**

A retrospective analysis of patients with PPF from 2015 through 2018 was done. Their sociodemographic, clinical, laboratory, ultrasonographic, endoscopic, and survival status data were collected for analysis. STATA 13 was used for analysis, the prevalence of varices, active schistosomiasis, and hepatitis B coinfection was determined. Cumulative mortality as a major outcome was also determined, and factors associated with increased risk of mortality were assessed by a logistic regression model.

**Results:**

In total, 250 participants were included in this analysis. Majority, 222 (88.8%; 95% CI: 84.2-92.4) had active *S. mansoni* infection, and 40 (16.0%; 95% CI: 11.6-21.1) had *S. mansoni*-HBV coinfection. Cumulatively, 39 (15.6%; 95% CI: 11.3-20.7) patients died, with most deaths, 31 (79.5%; 95% CI: 63.5-90.7) occurring within two years following the diagnosis of PPF (chi^2^ = 6.3; *p* = 0.012). The odds of mortality were independently associated with fishing (OR: 10.8; 95% CI: 2.2-52; *p* = 0.003), upper gastro intestinal bleeding (OR: 2.4; 95% CI: 1.1-5.4; *p* = 0.037), HBV coinfection (OR: 3.3; 95% CI: 1.2-91; *p* = 0.019), and ascites (OR: 3.3; 95% CI: 1.3-8.2; *p* = 0.010).

**Conclusions:**

In this, *S. mansoni* endemic area, varices, actives schistosomiasis, hepatitis B coinfection, and mortality are highly common. Screening for varices and initiation of prophylaxis, administration of praziquantel, and screening for hepatitis B should be part and parcel of care of these patients. The first two years of diagnosis, patients are at high risk of mortality; risk factors in this study should assist planning a closer follow-up of patients at risk of mortality to improve their long-term outcome.

## 1. Background

*Schistosoma mansoni* affects over 54 million people worldwide, and it is a major cause of high morbidity and mortality only second to malaria. More than 400 million people are at risk of infection especially in Sub-Saharan Africa (SSA) where more than 90% of the world's burden of *S. mansoni* is concentrated. Tanzania is the second most affected country after Nigeria [1], and *S. mansoni* is highly endemic in the Lake Zone part of the country especially among communities that are engaged in freshwater activities like fishing and rice farming [2, 3]. Due to the nature of these breadwinning activities, most people in Tanzania are at risk of chronic *S. mansoni* infection with serious clinical complications. Periportal fibrosis (PPF) commonly complicates chronic *S. mansoni* infection with high mortality due to bleeding varices [4, 5].

Community-based studies indicated that at least 30% of people infected *S. mansoni* have associated PPF. In the lake zone of Tanzania, up to 42% of patients studied for *S. mansoni* infection have been shown to have associated PPF [6, 7]. Similarly, hospital-based studies have also demonstrated a higher encounter of attendant varices among patients with PPF with high mortality. For instance in Saudi, 72% of patients who had PPF were found to have associated esophageal varices [8] before incident bleeding. However, it is estimated that about 30% of patients with PPF present late enough with fatal bleeding varices ending up with a mortality of 10-20% which might be even higher [9, 10]. A study from Bugando indicated that varices caused 70% of hematemesis with high mortality of more than 10% [10]. Studies elsewhere had reported even higher mortality rates up to 29% of PPF patients presenting with hematemesis [11, 12].

Previous studies had indicated that the mortality is increased among those patients who are coinfected with hepatitis B, severe liver fibrosis, and larger portal vein among others. Studies on factors associated with increased risk of mortality are still scarce especially in our setting where *S. mansoni* is still highly endemic. Understanding factors that increased the risk of mortality is important clinically in devising ways that can improve the outcome of this subgroup of patients.

## 2. Material and Methods

This was a clinic-based retrospective study involving all adult patients who were diagnosed to have periportal fibrosis between January 2015 and December 2018. The study was conducted at Bugando Medical Centre (BMC). BMC is a university teaching hospital that started operating under superspecialized units in 2015 with gastroenterology unit being one of the well-doing units in internal medicine. The unit has functioning endoscopic facilities and it works in collaboration with other departments including radiology and the main laboratory.

Patients with periportal fibrosis and cirrhosis are attended on daily bases in gastroenterology unit as common causes of portal hypertension. Routinely, abdominal ultrasound examination is done to determine the probable cause the portal hypertension and other details including portal vein and splenic diameter, and presence or absence of ascites is documented. Subsequently, endoscopic screening for attendant esophageal varices is done, and other additional tests including full blood count (FBP), hepatitis B surface antigen, liver function tests (LFT), and urine CCA or stool for *S. mansoni* are also done. Patients with PPF due to *S. mansoni* routinely get biannual praziquantel; propranolol for those with small varices and a series of endoscopic variceal ligation (EVL) in addition to propranolol is done among those with large varices.

A minimum sample size of 246 patients was estimated from the Kish-Lisle formula assuming cumulative mortality of 20% among patients with PPF as reported previously [9]. A clinic registry was used to identify all patients who were diagnosed to have PPF at BMC during the study period. Registration numbers were used to trace patients' files which were then reviewed by researchers for information of research interest. Demographic information, clinical presentation including abdominal distension, hematemesis, and passage of bloody stools; UTS details including splenic size, portal vein diameter (PVD), and ascites; test results for *S. mansoni*, FBP, AST, and ALT; upper endoscopy results; and survival status were recorded for analysis. Time in years from diagnosis of PPF to the enrolment into the study was recorded in years, and for patients who were dead, the time in years from diagnosis to death was noted.

Data were computerized using Epi data version 3.1, and STATA version 13 (Stata Corp LP, college station, TX) was used for analysis. Continuous variables were summarized as medians with interquartile range (IQR) while categorical variables were summarized as proportions with percentages. Death as the main outcome of patients was calculated and expressed as a percentage with 95% confidence interval (CI) and the distribution of deaths by time from diagnosis of PPF was determined. Odds ratio with 95% CI was calculated by univariate followed by a multivariate logistic regression model to assess the difference in degree of association between various factors with the mortality outcome. All factors with *p* < 0.25 on the univariate model were considered for subsequent inclusion in the multivariate model. In the final model factors with a *p* value < 0.05 were considered to have an independent association with the mortality outcome and the assessment for fitness of the model was done by Hosmer-Lemeshow.

### 2.1. Ethical Clearance

The permission to conduct and publish the findings from this study was sought from the Catholic University of Health and Allied Sciences (CUHAS)/BMC joint ethical committee with an ethical clearance certificate number 907/2019. The patients' information was handled by the researcher alone and their identifiers including names and registration numbers were not included in the final analysis to further maintain confidentiality.

## 3. Results

### 3.1. Sociodemographic, Clinical, and Laboratory Characteristics among 250 Participants

**Figure 1:**
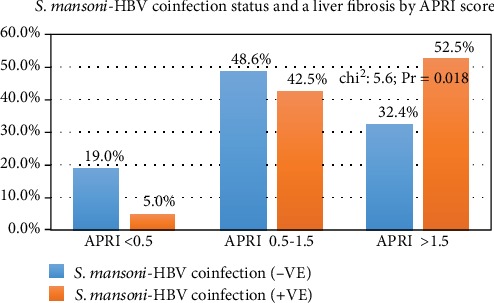
*S. mansoni*-HBV coinfection status and liver fibrosis by APRI score.

In total, 250 participants with *S. mansoni-*related PPF were analyzed. The majority, 180 (72%), were male participants with a median age of 41 (IQR: 35-51) years and a median postdiagnosis time of 2 (IQR: 1-3) years. Most participants, 197 (78.8%), were peasants and 24 (9.6%) were still engaging in fishing activities; however, the majority, 238 (95.6%), had contact with Lake Victoria water. Most patients, 171 (68.4%), presented with abdominal distension and 64 (25.6%) had a history of upper gastrointestinal bleeding. Of the studied participants, 108 (43.2%; 95% CI: 36.9-49.6%) were found to have esophageal varices, and 222 (88.8%; 95% CI: 84.2-.92.4) had active *S. mansoni* infection. In total, 44 (17.6%; 95% CI: 13.1-.22.9) had positive test for HBsAg and 40 (16.0%; 95% CI: 11.6-21.1) had *S. mansoni*-BV coinfection (Table 1). Patients with *S. mansoni*-hepatitis B coinfection were more likely to have a fibrosis APRI score > 1.5 (52.5% vs.32.4%, chi^2^: 5.6; Pr = 0.018) as summarized in (Table 2 and Figure 1).

### 3.2. Prevalence and Correlates of Mortality among 250 Study Participants with PPF

**Figure 2:**
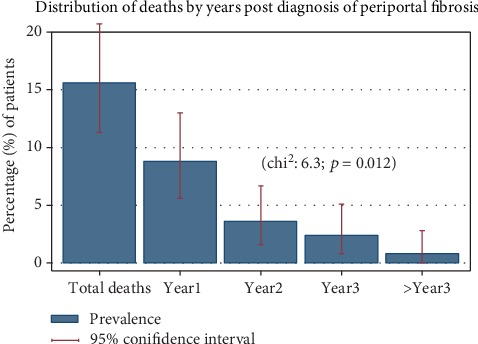
Distribution of deaths by years post diagnosis of periportal fibrosis.

**Figure 3:**
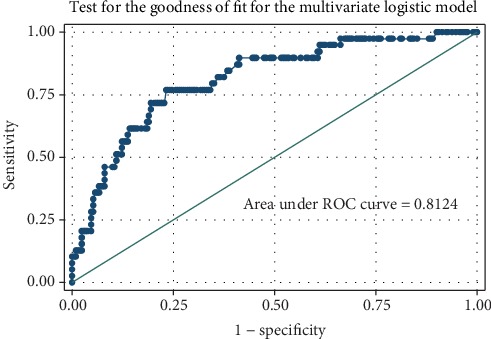
Test for the goodness of fit for the multivariate logistic model.

In this study, a total of 39 (15.6%; 95% CI: 11.3-20.7) were reported to pass on. Most of the reported deaths, 31(79.5%; 95% CI: 63.5-90.7), were more likely to occur within the first two years following diagnosis of PPF (chi^2^ = 6.3; *p* = 0.012) (Figure 2). The odds of mortality were independently associated with fishing (OR: 10.8; 95% CI: 2.2-52; *p* = 0.003), upper gastrointestinal bleeding (OR: 2.4; 95% CI: 1.1-5.4; *p* = 0.037), *S. mansoni*-HBV coinfection (OR: 3.3; 95% CI: 1.2-91; *p* = 0.019), and ascites (OR: 3.3; 95% CI: 1.3-8.2; *p* = 0.010). The difference in the distribution of other factors was not significant statically (Table 3), and the assessment for goodness of fit of the model did not demonstrate gross lack of fitness with area under the ROC curve of 0.8124 (Figure 3).

## 4. Discussion

The main objective of this study was to determine the prevalence and correlates of mortality among patients attended for periportal fibrosis at a tertiary level hospital in Mwanza Tanzania. In this study, 250 participants were analyzed where 108 (43.2%) were endoscopically found to have esophageal varices, 222 (88.8%) had active *S. mansoni* infection, and 44 (17.6%) were coinfected with hepatitis B. Over a median period of 2 (IQR: 1-3) years, 39 (15.6%) participants died and the risk of mortality was higher with fishing, hematemesis, ascites, and hepatitis B coinfection.

The prevalence of esophageal varices in our current study is similar to a prevalence rate of 45% reported from Uganda [13] and 47% reported in Sudan before praziquantel (PZQ) mass drug administration (MDA) [14]. However, the current prevalence rate is lower than the prevalence of 67% reported much earlier in a study from Sudan [15] and 72% that was reported from Saudi in 2011 [8]. Comparatively, the prevalence of esophageal varices in the current study is higher than the prevalence rate of 30% in Sudan the following year of MDA which was also associated with a significant reduction of PPF severity and *S. mansoni* infection [14]. The difference in prevalence of varices observed is more likely due to a discrepancy in the timing of screening for varices in these studies.

In our setting, active *S. mansoni* among patients with periportal fibrosis seems to be a very common encounter. The prevalence of active *S. mansoni* in the current study is similar to a previous report from Zambia (88.8% vs. 88%) [16]. However, it is slightly lower than earlier reports from Zambia (88.8% vs. 98%) where the diagnosis of active *S. mansoni* was based on rectal snip for Schistosoma ova [17]. Otherwise, the current prevalence is comparatively higher than what was reported from Kenya (88.8% vs. 72%) [18], 60% reported in 2014 from Bugando [10], 59.0% from Zimbabwe [19], and 53% reported from Sudan [14]. The current prevalence of active *S. mansoni* also is much higher than a prevalence of rate 34.5% reported recently from Bugando Medical Centre among PPF patients undergoing endoscopic variceal ligation for large varices [20].

Ongoing lake contact can partly explain the higher prevalence of active *S. mansoni* reported from Zambia [16] which is also similar to our current study where 238 (95.6%) of the studied participants had reported repeated contact with lake water. Moreover, the diagnosis of *S. mansoni* based on rectal snips could have included even those participants with nonviable ova in a study from Zambia [17]. Otherwise, PPF patients attending endoscopy unit for endoscopic variceal ligation are usually put on biannual praziquantel at Bugando [20], and probably those found with active *S. mansoni* infection represent a subgroup of patients with Schistosoma reinfection since this is a frequent phenomenon reported elsewhere following mass drug administration [21, 22].

The prevalence of *S. mansoni-*hepatitis B coinfection in this study is similar to concurrent infection of 15.8% reported by Aquino et al. from Brazil [23] and 16.1% reported in another study done by Berhe et al. in Ethiopia [24]. However the prevalence of *S. mansoni* and hepatitis B coinfection in the current study is comparatively lower than the prevalence rate of 19.6% reported earlier in 1991 by El-Sayed et al. from Egypt [25] and 58.4% reported recently by Du et al. from China [26]. But even with these difference on clinical grounds, this indicates that the Schistosoma endemic areas are also endemic for hepatitis B whose coinfection has been shown to have an even much severe liver disease and poorer outcome [27].

With the mortality rate reported in this study, 39 (15.6%) are comparatively similar to a previous study from Bugando among 124 patients with hematemesis. In this study, 91 participants underwent endoscopy and 13 (14.3%) died within 2 months of follow-up [10]. Otherwise the current mortality rate is higher than what was reported by Kheir et al. from Sudan (16.0% vs. 11.0%) in 2000 [14], and it is also much higher than the mortality of 6.25% among patients with periportal fibrosis who had rebleeding [28] and 5.9% reported from Uganda in 2007 [29]. Earlier studies from Sudan had reported a much higher prevalence of mortality of 25-29% following bleeding events [11, 12].

Mortality in this subgroup of patients partly denotes a delayed diagnosis of attendant varices and subsequent prevention of bleeding [30]. This is well supported in the current study that bleeding was reported in nearly 60% (*n* = 64) out of 108 of those with varices and the mortality was 2.4 times more likely among those with bleeding as compared to the nonbleeding counterparts (41.0% vs. 22.7%, OR: 2.4; *p* = 0.037). This is likely to occur among those with recurrent *S. mansoni* infection, in this case, those who are occupationally exposed to lake water [10] like the fishermen in this study. In previous studies, these patients were more likely to have severe periportal fibrosis on ultrasound assessment with a large portal vein diameter. But they were also more likely to have ascites similar to findings in our current study [10, 31–33] among others.

Coinfection with hepatitis B has been associated with higher odds of mortality previously in this subgroup of patients [31], similar to the findings in the current study. In an earlier study by Bassily et al., it was indicated that the mortality rate among *S. mansoni-*hepatitis B-coinfected patients was as high as 64.0% vs. 22% among those who were monoinfected [34]. This has been established previously as being due to severe inflammatory process with severe liver damage that occurs among patients who are *S. mansoni-*hepatitis B*-*coinfected patients which have also been shown to end up with increased morbidity and mortality [27, 35]. This is also supported partly by the finding that patients who were *S. mansoni*-hepatitis B coinfected were more likely to have higher fibrosis APRI scores of >1.5 as compared to their noncoinfected counterparts.

These findings are clinically important in our setting where *S. mansoni* is highly endemic suggesting that at any time, patients with PPF are at greater risk of having an ongoing active Schistosoma infection due to a possible high rate of reinfection. So apart from biannual PZQ, screening and treatment for active *S. mansoni* infection are advocated on routine bases. Giving PZQ is shown to reverse PPF and potentially reduces mortality [36, 37]. Routine screening for hepatitis B coinfection also is suggested by findings in this study. Patients with coinfection are likely to have a worse prognosis as compared to those who are monoinfected. Otherwise, patients with PPF should have an endoscopy done [32, 38] and prophylaxis started, with a close clinical follow-up particularly in the first two years [38]. Risk factors in this study are useful in the selection of patients at risk of mortality and on closer clinical follow-up to improve their outcomes.

This study is also liable to several limitations. It is a single-center study; thus, its findings may not be generable. Being retrospective, it lacks the temporal relation of events and a longitudinal assessment of the effect of treatment, and thus, a longitudinal study is recommended to address some of these issues. Otherwise, the information from this study is highly valuable in plans to address this important subgroup of people.

## Figures and Tables

**Table 1:** Study characteristics among 250 participants with Schistosoma periportal fibrosis.

Variable	Frequency	Percent (%) or median (IQR)
Sex		
Male	180	72.0
Female	70	28.0
Age (years)	250	41 (33-51)
Married		
Yes	215	86.0
No	35	14.0
Occupation		
Peasant	197	78.8
Fishing	24	9.6
Others	29	11.6
Lake water contact		
Yes	238	95.6
No	11	4.4
Alcohol use		
Yes	123	49.2
No	127	50.8
Abdominal distension		
Yes	171	68.4
No	79	31.6
Upper GIT bleeding		
Yes	64	25.6
No	186	74.4
Varices on OGD		
Yes	108	43.2
No	142	56.8
APRI > 1.5		
Yes	90	36.0
No	160	64.0
Years postdiagnosis	250	2 (1-3)

APRI: aspartate aminotransferase platelet count ratio Index; GIT: gastrointestinal tract; IQR: interquartile range; OGD: oesophagogastroduodenoscopy.

**Table 2:** Assessment of liver fibrosis by APRI scores among 250 study participants.

SHB coinfection	AST to platelet ratio index class			Total
	≤0.5	0.5-1.5	≥1.5	
No (*n* %)	40 (19.1)	102 (48.6)	68 (32.4)	210 (100.0)
Yes (*n* %)	2 (5.0)	17 (42.5)	21 (52.5)	40 (100.0)
Total (*n* %)	42 (16.8)	119 (47.6)	89 (35.6)	250 (100.0)

AST: aspartate aminotransferase; SHB: *Schistosoma mansoni*-hepatitis B virus.

**Table 3:** Factors associated with mortality among 250 patients with periportal fibrosis.

Variable	Mortality postdiagnosis		Unadjusted		Adjusted	
	No (*n* = 211)	Yes (*n* = 39)	OR (95% CI)	*p* value	OR (95% CI)	*p* value
Sex						
Female	63 (29.9)	7 (18.0)	1.0			
Male	148 (70.1)	32 (82.1)	1.9 (0.8-4.6)	0.133	1.2 (0.4-3.1)	0.728
Age	41 (33-51)	41 (35-54)	1.0 (0.9-1.03)	0.601		
Lake water contact						
No	10 (4.8)	1 (2.6)	1.0			
Yes	200 (95.2)	38 (97.4)	1.8 (0.2-15.3)	0.546		
Occupation						
Fishing	13 (6.2)	11 (28.2)	5.9 (2.4-14.6)	<0.001	10.8 (2.2-52.0)	0.003
Peasant	173 (82.0)	24 (61.5)	0.3 (0.2-0.7)	0.005	1.1 (0.3-4.1)	0.844
Upper GIB						
No	163 (77.3)	23 (58.0)	1.0			
Yes	48 (22.7)	16 (41.0)	2.4 (1.1-4.8)	0.018	2.4 (1.1-5.4)	0.037
*S. mansoni*						
Negative	20 (9.5)	8 (20.5)	1.0			
Positive	191 (90.5)	31 (79.5)	0.4 (0.2-1.0)	0.050	0.2 (0.1-0.6)	0.005
HBV coinfection						
No	183 (86.7)	27 (69.2)	1.0			
Yes	28 (13.3)	12 (30.8)	2.9 (1.3-6.3)	0.008	3.3 (1.2-9.1)	0.019
PTC (10^3^/*μ*l)	89 (59-126)	93 (71-185)	1.0 (0.9-1.0)	0.680		
PVD (cm)	1.5 (1.4-1.7)	1.5 (1.4-1.8)	1.8 (0.8-4.1)	0.117	2.4 (0.7-8.4)	0.145
APRI	1.1 (0.7-2.2)	1.0 (0.6-2.0)	0.9 (0.7-1.1)	0.488		
SPD (cm)	17 (15-18)	17 (15.19)	1.1 (0.9-1.2)	0.289		
Ascites						
No	101 (47.9)	9 (23.1)	1.0			
Yes	110 (52.1)	30 (76.9)	3.1 (1.3-6.7)	0.006	3.3 (1.3-8.2)	0.010
Varices on OGD						
No	116 (55.0	25 (64.1)	1.0			
Yes	95 (45.0)	14 (35.9)	0.6 (0.3-1.3)	0.293		

CI: confidence interval; HBV: hepatitis B virus; OR: odds ratio; PTC: platelet count; SPD: splenic diameter; PVD: portal vein diameter; UGIB: upper gastro intestinal bleeding.

## Data Availability

No data were used to support this study.
